# Contemporary diagnosis and management of vestibular migraine: a systematic review of conventional and CGRP-targeted therapies

**DOI:** 10.3389/fneur.2026.1951810

**Published:** 2026-10-27

**Authors:** Saleh Alabood, Laila Aldokhail, Enar Alotaibi, Mayar Alsaqr, Anwar Alshehri, Essa Bakry

**Affiliations:** 1 Department of Otorhinolaryngology-Head and Neck Surgery, Security Forces Hospital, Riyadh, Saudi Arabia; 2 College of Medicine, Princess Norah Bint Abdulrahman University, Riyadh, Saudi Arabia; 3 Department of Otolaryngology-Head and Neck Surgery, King Saud University Medical City, King Saud University, Riyadh, Saudi Arabia

**Keywords:** CGRP-targeted therapy, erenumab, galcanezumab, neuro-otology, rimegepant, systematic review, vertigo, vestibular migraine

## Abstract

**Background:**

Vestibular migraine (VM) is a common cause of recurrent episodic vertigo, and it is associated with significant disability from recurrent vestibular and migraine symptoms. While there are a number of standard preventive treatments, their effectiveness is quite variable, and new treatments which target calcitonin gene-related peptide (CGRP) have been identified as potential treatments. The purpose of this systematic review was to assess the state of the art in the diagnosis of vestibular migraine and to compare the efficacy of conventional pharmacological treatments with the new CGRP-targeted treatments.

**Methods:**

A systematic literature review was performed to find studies that assessed the diagnosis and pharmacological treatment of vestibular migraine. Randomized controlled trials, observational studies, comparative studies, retrospective analysis, cross-sectional studies, and case reports of patients with definite or probable vestibular migraine were included. Study characteristics, diagnostic methods, therapeutic interventions, clinical outcomes, and treatment efficacy were qualitatively extracted and synthesized.

**Results:**

21 studies were included with a total of over 12,000 participants. The evidence comprised predominantly adult VM populations together with one large pediatric multicenter study. Among adult studies, participants were predominantly middle-aged women. The Bárány Society/ICHD criteria were used as a primary basis for the diagnostic evaluation, with selective tests of the vestibular system and neuroimaging to rule out alternative diagnoses. conventional preventive therapies were associated with reductions in vertigo frequency, headache burden, and dizziness-related disability. Anti-CGRP therapies including erenumab, fremanezumab, galcanezumab, rimegepant, and ubrogepant, were associated with clinically meaningful improvements in vertigo frequency, monthly dizziness days, disability scores, headache outcomes, and patient-reported quality of life.

**Conclusion:**

A multimodal approach to the clinical evaluation of vestibular migraine is supported by current evidence, and both traditional preventive treatments and CGRP-targeted treatments have demonstrated benefit across vestibular and migraine-related outcomes. CGRP-targeted therapies represent a promising emerging therapeutic option, particularly for patients with persistent or refractory symptoms. however, the limited randomized evidence and scarcity of direct head-to-head comparisons with conventional therapies preclude firm conclusions regarding their comparative effectiveness. **Systematic review registration:** identifier: CRD420261427685.

## 1 Introduction

Vestibular migraine (VM) is a common, often underrecognized neurological disorder and a leading cause of episodic vertigo and dizziness (1, 2). characterized by recurrent episodes of vestibular symptoms in individuals with active or previous history of migraine. These episodes may last from minutes to days and are often accompanied by migraine features, including (headache, photophobia, phonophobia and/or visual aura) (3). The prevalence of this disorder is estimated at approximately 1 to 3% of the general population and can make up a significant percentage of patients attending neurology and otolaryngology clinics with unexplained vertigo (4). Vestibular migraine predominantly affects women and most and commonly seen in the fourth and fifth decade of life, causing a significant burden on quality of life, daily functioning and health care utilization (5).

Although VM is relatively common, diagnosis of vestibular migraine is difficult, as there is currently no specific diagnostic biomarker (5). The primary method of diagnosis is clinical evaluation using standardized diagnostic criteria developed jointly by the Bárány Society and the International Headache Society (6). The clinical presentation is often heterogeneous and may overlap with other vestibular conditions like Ménière's disease, benign paroxysmal positional vertigo (BPPV), and persistent postural-perceptual dizziness (PPPD), which can contribute to diagnostic uncertainty, delayed diagnosis, or misdiagnosis (6). Consequently, patients may undergo extensive investigations before an accurate diagnosis is established, highlighting the need for improved diagnostic strategies and greater clinical recognition of the diverse manifestations of VM.

Treatment of vestibular migraine has largely been extrapolated from established migraine therapies because relatively few high-quality randomized controlled trials have specifically evaluated vestibular outcomes in patients with VM (7, 8). The conventional approach to management involves lifestyle modification, trigger avoidance, vestibular rehabilitation, and pharmacological prophylaxis (prevention) using drugs such as beta-blockers, calcium channel blockers, antidepressants and antiepileptics (7). These methods are effective for many patients, but there are variable responses to treatment, and side effects often make it difficult for patients to follow treatment for long periods of time. In addition, there is significant clinical variation in treatment practice due to the absence of standard treatment algorithms (7, 8).

There have been recent advances in understanding migraine physiology, which has identified calcitonin gene-related peptide (CGRP) as a major player in migraine mechanisms and has resulted in the development of CGRP-targeted therapeutics: monoclonal antibodies and small molecule CGRP receptor antagonists (gepants) (9, 10). These agents have shown good efficacy and tolerability in the prevention and acute treatment of migraine and have been interesting as therapeutic targets for vestibular migraine (9). Early clinical trials and observational studies suggest that CGRP-targeted therapies may reduce both headache burden and vestibular symptoms. However, the available evidence remains limited and heterogeneous. Consequently, the precise role of these novel therapies within contemporary VM treatment algorithms remains uncertain and requires further systematic evaluation.

Given the evolving landscape of VM diagnosis and treatment, an updated and comprehensive synthesis of the available evidence is warranted. Although previous reviews have addressed individual aspects of VM diagnosis, conventional management, or emerging CGRP-targeted treatments, they have largely examined these domains separately, while earlier reviews of CGRP-targeted therapies were based on a limited and rapidly evolving evidence base. Consequently, there remains a need for an integrated evaluation of contemporary diagnostic approaches alongside the full spectrum of established and emerging therapeutic strategies. This integration is particularly important in VM because no validated disease-specific biomarker exists, and the interpretation of therapeutic outcomes depends on accurate clinical classification and the exclusion of overlapping vestibular disorders. Variability in diagnostic criteria and diagnostic work-up may consequently influence patient selection, reported treatment responses, and the generalizability of therapeutic findings. Accordingly, this systematic review aims to evaluate current diagnostic approaches for VM and systematically assess the efficacy and safety of conventional and emerging CGRP-targeted therapies. By integrating evidence across these interdependent diagnostic and therapeutic domains, this review seeks to provide a contemporary synthesis of clinical practice, identify important gaps in the literature, clarify the emerging role of CGRP-targeted therapies, and inform future research priorities and evidence-based clinical decision-making.

## 2 Methodology

### 2.1 Study design and reporting guidelines

The Preferred Reporting Items for Systematic Reviews and Meta-Analyses (PRISMA) 2020 statement was followed to ensure the identification, selection, appraisal, and synthesis of available evidence is transparent, reproducible, and methodologically rigorous. The review protocol was designed before the selection of the studies, and all the steps of the review process were done systematically to reduce bias. The review protocol was prospectively registered in the International Prospective Register of Systematic Reviews before formal study selection and data extraction.

### 2.2 Literature search strategy

A comprehensive literature search was conducted in electronic databases (PubMed/MEDLINE, Scopus, Web of Science, Embase, and the Cochrane Library) from database inception through June 25, 2026. The search strategy included: controlled vocabulary terms and free-text keywords for vestibular migraine, diagnosis, management, treatment, calcitonin gene-related peptide (CGRP), CGRP monoclonal antibodies, gepants, vestibular disorders, migraine prophylaxis, and associated synonyms. Boolean operators (“AND” and “OR”) were employed to refine the search strategy and the reference lists of all eligible studies and relevant review articles were manually screened to identify additional eligible studies that may not have been located during the electronic search.

### 2.3 Eligibility criteria

The studies included those that assessed the clinical outcomes of patients with a diagnosis of vestibular migraine based on established diagnostic criteria, without restriction by age, explored diagnostic and therapeutic strategies, or examined treatment efficacy, safety, and diagnostic performance. Randomized controlled trials, non-randomized interventional studies, prospective and retrospective cohort studies, case-control studies and observational studies were included. Case reports were also considered when they provided clinically relevant evidence regarding emerging therapies, uncommon manifestations, or detailed diagnostic assessments that were not otherwise represented in higher-level studies. Given the emerging nature of CGRP-targeted therapy in VM and the limited evidence available for certain diagnostic and therapeutic questions, inclusion of a broader range of study designs was considered necessary to capture the contemporary evidence base. However, evidence from case reports was interpreted descriptively and was not considered equivalent in evidentiary strength to controlled or comparative studies. Studies including exclusively conference abstracts without full text, editorials, letters, narrative reviews, systematic reviews, animal studies and studies published in languages other than English were excluded.

### 2.4 Study selection

After this electronic search, all the retrieved citations were imported into reference management software and duplicate records were removed. All identified studies were screened by two independent reviewers using the pre-defined eligibility criteria by reviewing the title and abstract. Potentially eligible articles were then identified, and reports were sought for full-text retrieval and independently evaluated for inclusion. Disagreements about the eligibility of studies resolved through consensus and discussion and, when required, a third reviewer.

### 2.5 Data extraction

Two reviewers extracted data from the literature independently, using a standardized data extraction form. Information extracted comprised the characteristics of the study, the year of publication, the country of origin, the study design, the number of participants, their age and sex distributions, diagnostic criteria for vestibular migraine, comparator groups, therapeutic interventions, follow-up period, outcome measures, treatment efficacy, adverse events, and the main findings reported by each study. Discrepancies in the information retrieved were checked against the original articles and consensus was reached.

### 2.6 Outcome measures

The outcomes of interest were predefined and categorized into diagnostic and therapeutic domains. The primary diagnostic outcomes included the application of established diagnostic criteria for vestibular migraine and the clinical utility of vestibular, audiological, and radiological assessments in supporting the diagnosis or excluding alternative vestibular disorders. The primary therapeutic outcomes included changes in vertigo or vestibular symptom frequency and severity, monthly dizziness or vestibular symptom days, and dizziness-related disability measured using validated instruments such as the Dizziness Handicap Inventory (DHI) and Vestibular Migraine Patient Assessment Tool and Handicap Inventory (VM-PATHI).

Secondary outcomes included headache or migraine frequency and severity, migraine-related disability measured using instruments such as the Migraine Disability Assessment (MIDAS) and Headache Impact Test-6 (HIT-6), patient-reported global improvement, quality of life, psychological outcomes, auditory symptoms, changes in vestibular function tests, treatment response rates, adverse events, and treatment tolerability. Given the heterogeneity in outcome definitions and measurement instruments across the included studies, outcomes representing comparable clinical domains were synthesized narratively.

### 2.7 Risk of bias and certainty of evidence

Validated risk-of-bias and critical appraisal tools appropriate to each study design were used to evaluate the methodological quality of the included studies. The Cochrane Risk of Bias 2 (RoB 2) tool was used for randomized controlled trials, while the corresponding Joanna Briggs Institute (JBI) critical appraisal checklists were used for quasi-experimental, cohort, analytical cross-sectional studies, and case reports. Two reviewers independently performed the assessments, and disagreements were resolved through discussion and consensus.

The certainty of evidence for the main therapeutic outcomes was assessed using the Grading of Recommendations Assessment, Development and Evaluation (GRADE) approach, considering risk of bias, inconsistency, indirectness, imprecision, and publication bias. Certainty was classified as high, moderate, low, or very low.

### 2.8 Data synthesis

A qualitative narrative synthesis was performed, with findings organized according to diagnostic approaches, conventional pharmacological therapies, and CGRP-targeted therapies. The feasibility of meta-analysis was assessed both across the overall evidence base and within clinically comparable treatment subsets. However, quantitative pooling was not performed because potentially comparable studies remained heterogeneous in study design, interventions and dosing regimens, comparator groups, patient characteristics, outcome definitions and measurement instruments, and follow-up duration. This was particularly evident among CGRP-targeted studies, which evaluated different agents and included both acute and preventive treatments, and among conventional therapy studies, which included multiple pharmacological classes and different comparators. These clinical and methodological differences limited the ability to combine the results reliably. Accordingly, the findings were synthesized narratively by comparing the direction, magnitude, and consistency of reported treatment effects across studies.

## 3 Results

### 3.1 The general characteristics of the studies included

A total of 21 studies exploring vestibular migraine were included (Figure 1), (9–29), covering a variety of study designs, populations and interventions. The available evidence was methodologically heterogeneous and included randomized controlled trials, prospective and retrospective observational studies, comparative studies, case reports, retrospective chart reviews, a multicenter cross-sectional study, and a multicenter open-label study. The majority of the included studies enrolled adults with definite or probable vestibular migraine. A smaller number of studies specifically investigated patients with refractory vestibular migraine, those with migraine accompanied by auditory symptoms, or broader migraine populations in which vestibular migraine constituted a predefined subgroup.

**Figure 1:**
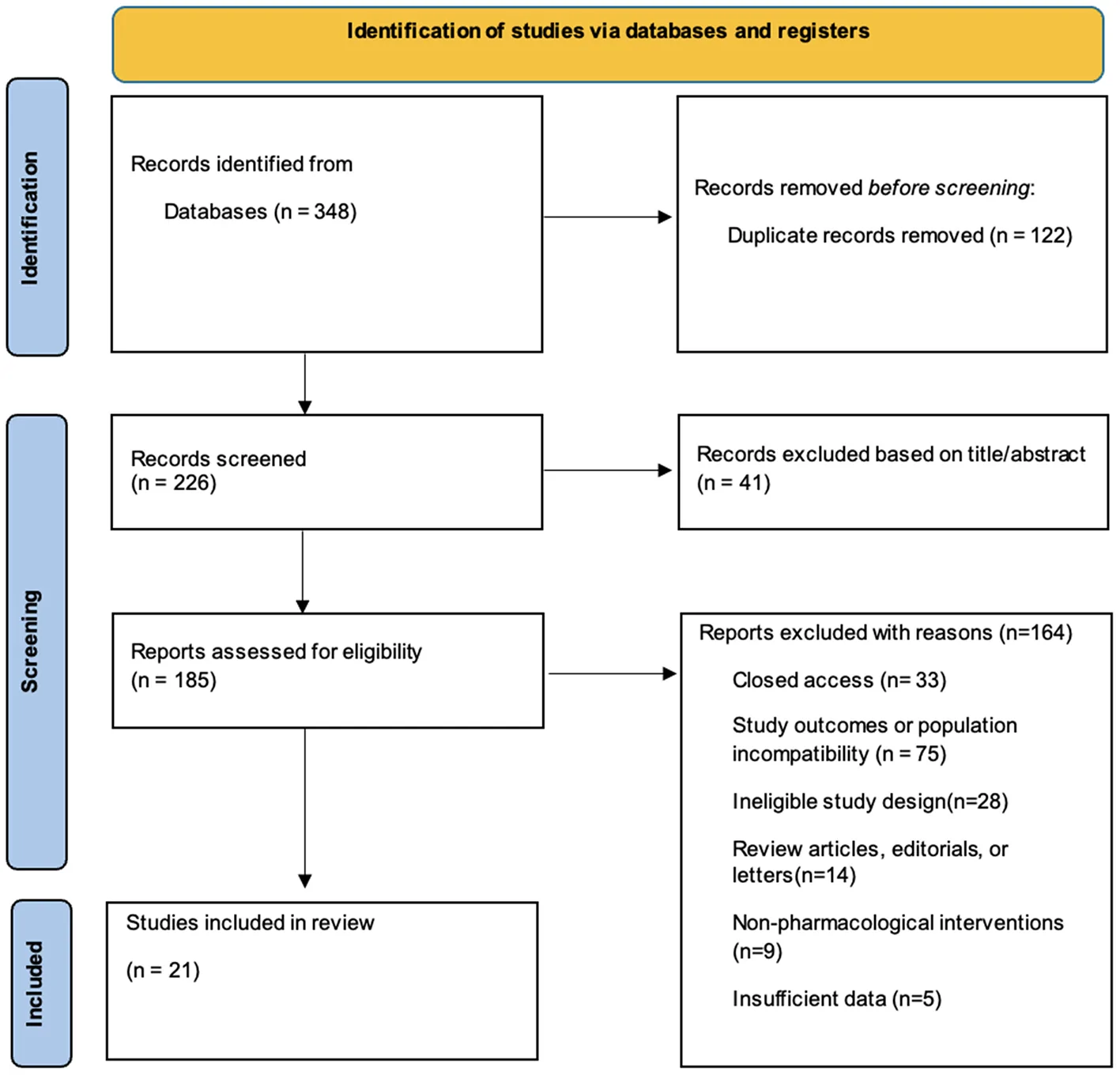
PRISMA flow diagram for study selection.

Epidemiological evidence was provided by one large multicenter cross-sectional study of more than 10,000 children with headache disorders, which characterized migraine-associated symptom patterns in a pediatric population (21). The remaining studies predominantly evaluated adult populations with definite or probable VM, with sample sizes ranging from single-patient case reports to approximately 20–80 participants in most interventional studies. The reported mean age in the adult studies was predominantly within the 4th and 5th decade of life (41–47 years), while the median age in the pediatric study was 13 years. In studies reporting sex distribution, females participants predominated, with about 66–100% of the participants being female, which is consistent with the known female predominance of vestibular migraine.

The main aims of the studies included were to evaluate preventive pharmacological therapies, to assess the efficacy and safety of calcitonin gene-related peptide (CGRP) targeted therapies, to compare conventional prophylactic drugs, to explore diagnostic strategies and to describe migraine symptom profiles. Conventional preventive treatments were β-blockers, calcium-channel blockers, anticonvulsants, antidepressants, acetazolamide, combinations of cinnarizine and dimenhydrinate, nimodipine, and botulinum toxin. Targeted biological treatments included erenumab, fremanezumab, and galcanezumab, while four studies evaluated treatment with gepants. (9, 10, 14, 16, 19, 20, 22, 28).

Follow-up ranged from acute assessment for 48 h in abortive treatment studies to long-term follow-up up to 18 months in preventive therapy studies. Outcome measures included patient-reported global improvement, adverse events, imaging findings, vestibular function, symptom-specific scales [Dizziness Handicap Inventory (DHI), Migraine Disability Assessment (MIDAS), Vestibular Migraine Patient Assessment Tool and Handicap Inventory (VM-PATHI), Vertigo Severity Score (VSS), Headache Impact Test−6 (HIT-6), Korean Dizziness Handicap Inventory (KDHI), University of California, Los Angeles Dizziness Questionnaire (UCLA-DQ), visual analog scales (VAS)], and vestibular physiological testing (Table 1).

**Table 1:** General characteristics of included studies.

Study	Country	Study design	Sample size	Population	Mean age (years)	Aim	Intervention/ Comparator	Intervention characteristics	Follow-up	Outcomes assessed	Main findings
Russo et al. (16)	Italy	Prospective observational cohort	50	Adults with vestibular migraine	45.0 ± 13.3	To evaluate the efficacy of anti-CGRP monoclonal antibodies in vestibular migraine	Erenumab, Fremanezumab, Galcanezumab	Anti-CGRP monoclonal antibodies administered according to routine clinical practice	18 months	Vertigo frequency, headache frequency, MIDAS, monthly dizziness days	Anti-CGRP monoclonal antibodies produced sustained and clinically meaningful reductions in vertigo, headache frequency, and migraine-related disability in patients with vestibular migraine.
Hoskin et al. (10)	USA	Retrospective cohort	28 (25 completed follow-up)	Vestibular migraine patients prescribed CGRP medications	NR	To evaluate the effectiveness of CGRP-targeted medications in vestibular migraine	Erenumab, Galcanezumab, Fremanezumab, Ubrogepant	Retrospective assessment of clinical response	Variable	Improvement in vestibular symptoms, migraine symptoms, treatment compliance	Most patients experienced clinically meaningful improvement in vestibular and migraine symptoms following CGRP-targeted therapy in routine clinical practice.
Lovato et al. (28)	Italy	Retrospective comparative study	23	Vestibular migraine refractory to conventional therapy	45.2 ± 7.8	To compare anti-CGRP monoclonal antibodies with conventional preventive therapies	Erenumab vs. propranolol/ flunarizine/ valproate	Erenumab 140 mg every 28 days compared with previous conventional therapy	Mean 26.4 weeks	DHI, migraine days, videonystag -mography findings	Erenumab was associated with greater improvement in vestibular disability, migraine frequency, and vestibular findings than the previous conventional preventive treatment in this retrospective comparison.
Inui et al. (13)	Japan	Case report	1	Vestibular migraine	42	To assess vestibular examinations before and after erenumab treatment	Erenumab with previous migraine medications	Monthly erenumab following conventional therapy	1 year	Vestibular examinations, MRI, DHI, MIDAS, HIT-6	Erenumab markedly improved clinical symptoms despite persistent abnormalities on vestibular testing, indicating that symptomatic improvement may occur without normalization of objective vestibular findings.
Kwon et al. (9)	South Korea	Prospective observational study	20	Vestibular migraine	44.9 ± 14.7	To evaluate galcanezumab and fremanezumab in vestibular migraine	Galcanezumab vs. Fremanezumab	Monthly injections for ≥2 months	≥2 months	KDHI, monthly dizziness days, VAS, PGIC, functional level, monthly headache days	Both galcanezumab and fremanezumab significantly reduced dizziness, headache burden, and vestibular disability while demonstrating good tolerability.
Ihara et al. (25)	Japan	Single-center retrospective observational study	19	Adults with migraine receiving first erenumab treatment	45.2 ± 12.9	To evaluate real-world effectiveness, safety, and patient satisfaction with erenumab	Erenumab	Monthly erenumab 70 mg	3 months	Monthly migraine days, responder rate, migraine-associated symptoms, satisfaction, adverse events	Erenumab effectively reduced migraine burden and associated symptoms with high patient satisfaction and an acceptable safety profile in routine practice.
Sharon et al. (14)	USA	Double-blind randomized placebo-controlled trial	40 randomized (38 analyzed)	Adults with vestibular migraine	NR	To evaluate the efficacy of galcanezumab in vestibular migraine	Galcanezumab vs. placebo	240 mg loading dose followed by 120 mg monthly for 2 months	3 months	VM-PATHI, DHI, definite dizzy days, safety	Galcanezumab significantly improved vestibular migraine symptoms and dizziness-related disability compared with placebo without major safety concerns.
Goto et al. (18)	Japan	Case report	1	Vestibular migraine with tinnitus and hyperacusis	44	To evaluate the effect of galcanezumab on vestibular migraine-associated auditory symptoms	Amitriptyline followed by galcanezumab	Amitriptyline, Kampo medicine, then monthly galcanezumab	6 months	Hyperacusis, HIT-6, migraine frequency, dizziness	Galcanezumab successfully controlled vestibular migraine and associated auditory symptoms after inadequate response to conventional therapy.
Choi et al. (27)	South Korea	Multicenter prospective open-label study	850 migraine patients (255 VM; 131 completed VM follow-up)	Migraine including vestibular migraine	41 ± 12	To compare nimodipine, topiramate, and combination therapy in migraine and vestibular migraine	Nimodipine vs. Topiramate vs. Combination	Oral preventive therapy	3 months	Headache frequency, DHI, UCLA-DQ, dizziness days, MIDAS, HIT-6	Nimodipine, topiramate, and combination therapy all significantly improved vestibular migraine outcomes, with no clear superiority for dizziness control.
Staab et al. (26)	USA	Double-blind randomized placebo-controlled trial	222 enrolled (134 treated attacks)	Adults with vestibular migraine	42.3 ± 11.7	To determine the efficacy of rizatriptan for acute vestibular migraine attacks	Rizatriptan vs. placebo	Oral rizatriptan 10 mg for up to three attacks	Up to 48 h per treated attack	Vertigo, dizziness, headache, quality of life, adverse events	Rizatriptan provided limited benefit for acute vestibular symptoms despite modest improvement in headache-related outcomes.
Salviz et al. (15)	Turkey	Prospective randomized controlled trial	64 randomized (52 completed)	Adults with vestibular migraine	NR	To compare the efficacy of venlafaxine and propranolol for vestibular migraine prophylaxis	Venlafaxine vs. Propranolol	Venlafaxine or propranolol administered for preventive treatment	4 months	DHI, Vertigo Severity Score (VSS), vertigo attack frequency, BAI, BDI, adverse events	Venlafaxine and propranolol were similarly effective for vestibular migraine prophylaxis, although venlafaxine provided additional improvement in depressive symptoms.
Lepcha et al. (23)	India	Randomized controlled trial	48	Adults with migrainous vertigo	NR	To evaluate flunarizine for prophylaxis of migrainous vertigo	Flunarizine plus betahistine vs. betahistine alone	Flunarizine 10 mg daily with symptomatic treatment compared with symptomatic treatment alone	12 weeks	Vertigo frequency, vertigo severity, headache frequency, headache severity, adverse events	Flunarizine significantly reduced the frequency and severity of vertigo compared with symptomatic treatment alone.
Gode et al. (11)	Turkey	Randomized comparative trial	30	Adults with migrainous vertigo	38.5 ± NR	To evaluate topiramate efficacy and compare 50 mg/day with 100 mg/day	Topiramate 50 mg/day vs. 100 mg/day	Oral topiramate prophylaxis at two dosage regimens	6 months	Vertigo frequency, vertigo severity (VAS), headache frequency, headache severity (VAS), adverse events	Both topiramate doses effectively reduced vertigo and headache frequency, while the lower dose demonstrated better tolerability.
Gorur et al. (12)	Turkey	Prospective comparative study	60	Adults with vestibular migraine	45.9 ± 15.3	To evaluate oral preventive medications with or without botulinum toxin A	Oral prophylaxis ± botulinum toxin A	Propranolol, flunarizine, or amitriptyline with or without botulinum toxin A	NR	DHI, MIDAS, vertigo attack frequency, adverse events	Adding botulinum toxin A to oral prophylaxis enhanced improvement in migraine disability and vertigo frequency compared with oral therapy alone.
Teggi et al. (24)	Italy	Prospective controlled comparative study	33 (22 treatment; 11 control)	Adults with vestibular migraine	NR	To evaluate the efficacy of cinnarizine/dimenhydrinate (Arlevertan®) for vestibular migraine prophylaxis	Arlevertan® vs. lifestyle measures	Cinnarizine 20 mg + dimenhydrinate 40 mg twice daily in repeated monthly cycles compared with lifestyle modification alone	6 months	Vertigo attacks, headache attacks, responder rate, adverse events	Cinnarizine/dimenhydrinate significantly reduced vertigo and headache attacks compared with lifestyle modification alone.
Dominguez-Duran et al. (29)	Spain	Multicenter prospective observational study	31	Patients with vestibular migraine	46.8	To evaluate the effectiveness of different prophylactic medications in vestibular migraine	Acetazolamide, Amitriptyline, Flunarizine, Propranolol, Topiramate	Physician-selected preventive therapy according to clinical practice	5 weeks	Vestibular symptom intensity, headache intensity, monthly crises, adverse events	Physician-selected preventive therapies significantly improved vestibular symptoms and headache regardless of the medication prescribed.
Salmito et al. (17)	Brazil	Retrospective clinical chart review	81	Patients attending a vestibular migraine clinic	46	To compare the diagnostic performance of 2001 and 2012 vestibular migraine criteria and evaluate prophylactic treatment outcomes	Various prophylactic medications	Individualized prophylactic therapy including antidepressants, anticonvulsants, calcium-channel blockers and β-blockers	NR	Diagnostic classification, headache VAS, dizziness VAS, therapeutic response	Preventive treatment effectively reduced headache and dizziness irrespective of the diagnostic criteria used for vestibular migraine.
Shao et al. (19)	China	Multicenter prospective self-controlled cohort	33	Adults with vestibular migraine	45.2	To evaluate the preventive efficacy of rimegepant in vestibular migraine	Rimegepant	Oral rimegepant 75 mg every other day as preventive therapy	4 weeks	Monthly vestibular symptom days, headache days, DHI, VSS-SF, GAD-7, PHQ-9, HIT-6, ABC, hearing function	Preventive rimegepant rapidly and significantly reduced vestibular symptoms, headache frequency, psychological distress, and disability while improving quality of life and hearing outcomes.
Chu et al. (20)	China	Prospective single-arm clinical study	18	Definite or probable vestibular migraine	39.6 ± 14.3	To evaluate the acute efficacy of rimegepant for vestibular migraine attacks	Rimegepant	Oral rimegepant 75 mg administered during acute vestibular migraine attacks	48 h	Vertigo, dizziness, nausea, photophobia/ phonophobia, headache severity, VAS, adverse events	Acute rimegepant provided rapid and sustained relief of vestibular migraine symptoms with excellent tolerability and no reported adverse events.
Li et al. (22)	China	Retrospective cohort study	86	Adults with vestibular migraine treated for acute attacks	Median 48.0 years	To identify predictors of acute response to rimegepant in vestibular migraine	Rimegepant	Oral rimegepant 75 mg orally disintegrating tablet for acute attacks	2 h after each treated attack	Vertigo relief, vertigo NRS, complete vertigo freedom, remission, predictors of treatment response	Rimegepant was highly effective for acute vestibular migraine, with younger age and better hearing predicting greater treatment response.

### 3.2 Risk of bias and certainty of evidence

The methodological quality of the included studies varied according to study design. Methodological limitations were primarily identified among randomized and non-randomized interventional studies, while the analytical cross-sectional studies and case reports generally met the applicable JBI criteria. Case reports were considered descriptive supportive evidence rather than evidence of comparative treatment effectiveness. The detailed risk-of-bias and methodological quality assessment is presented in Supplementary Figure 1.

The certainty of evidence for the major therapeutic outcomes was generally low (Table 2). For conventional preventive therapies, certainty was limited by risk-of-bias concerns and heterogeneity. For preventive CGRP-targeted therapies and acute pharmacological treatment, certainty was primarily limited by heterogeneity and imprecision.

**Table 2:** GRADE summary of certainty of evidence for major therapeutic outcomes in vestibular migraine.

Major therapeutic outcome	Evidence base	Risk of bias	Inconsistency	Indirectness	Imprecision	Publication bias	Overall certainty
Conventional preventive therapy: vestibular symptoms/disability	Randomized and non-randomized studies (11, 12, 15, 23, 24, 27, 29)	Serious	Serious	Not serious	Not serious	Cannot be reliably assessed	Low
Conventional preventive therapy: headache/migraine outcomes	Randomized and non-randomized studies (11, 12, 15, 23, 24, 27, 29)	Serious	Serious	Not serious	Not serious	Cannot be reliably assessed	Low
Preventive CGRP-targeted therapy: vestibular symptoms/disability	Randomized and observational studies (9, 10, 14, 16, 19, 28)	Not serious	Serious	Not serious	Serious	Cannot be reliably assessed	Low
Preventive CGRP-targeted therapy: headache/migraine outcomes	Randomized and observational studies (9, 10, 14, 16, 19, 28)	Not serious	Serious	Not serious	Serious	Cannot be reliably assessed	Low
Acute pharmacological treatment: vestibular symptom improvement	Randomized and non-randomized studies (20, 22, 26)	Not serious	Serious	Not serious	Serious	Cannot be reliably assessed	Low

### 3.3 Diagnostic assessment of the vestibular migraine

Diagnostic assessment across all included studies comprised a combination of clinical diagnostic criteria, radiological and vestibular functional assessment to help establish the diagnosis of vestibular migraine and rule out other potential vestibular disorders. Across the included evidence, VM remained fundamentally a clinical diagnosis based on established diagnostic criteria. The main purpose of imaging modalities, including contrast-enhanced brain MRI and gadolinium-enhanced inner ear MRI was to rule out structural or peripheral vestibular pathology rather than to make a definite diagnosis. Vestibular functional tests often included videonystagmography (VNG), caloric testing, cervical and ocular vestibular evoked myogenic potentials (cVEMP and oVEMP), video head impulse testing (vHIT), posturography, audiometry and standardized patient-reported outcome measures, including the Dizziness Handicap Inventory (DHI), Migraine Disability Assessment (MIDAS), and HIT-6 questionnaire (13, 18, 28).

Overall, the findings indicated that objective vestibular abnormalities were variable and poorly correlated with clinical improvement of symptoms. Although normalization of VNG abnormalities has been reported after anti-CGRP therapy, other studies found that even though dizziness and headache were significantly improved, the VNG remained abnormal, indicating that symptomatic improvement could be achieved without measurable changes in peripheral vestibular function (13, 28). A Comprehensive vestibular assessment also facilitated the exclusion of alternative vestibular disorders, particularly in patients presenting with auditory symptoms such as tinnitus and hearing loss, thereby supporting the diagnosis of vestibular migraine before initiating disease-specific treatment (18).

Diagnostic criteria were also evaluated and differences between historical and contemporary classification systems were found. When the 2001 and 2012 Bárány/ICHD criteria were compared, the new criteria diagnosed fewer patients and increased the specificity without impacting the subsequent response to preventive treatment (17). In general, the evidence provided shows the importance of a multimodal diagnostic strategy that combines standard clinical criteria with a selective use of vestibular and radiological tests to exclude alternative diagnoses instead of using only one objective test for the diagnosis (Table 3).

**Table 3:** Summary of studies reporting diagnostic approaches for vestibular migraine.

Study	Diagnostic method	Diagnostic findings	Comparator/ Reference	Main diagnostic outcome	Author's conclusion
Lovato et al. (28)	Contrast-enhanced MRI, Videonystagmography (VNG), Dizziness Handicap Inventory (DHI)	MRI excluded structural causes; VNG abnormalities improved significantly following anti-CGRP therapy	Pre- vs. post-treatment; conventional therapy comparison	Normalization of vestibular findings after anti-CGRP therapy	Objective vestibular abnormalities improved only after anti-CGRP treatment
Inui et al. (13)	Pure-tone audiometry, Caloric test, cVEMP, oVEMP, Gadolinium-enhanced inner ear MRI, DHI, MIDAS, HIT-6	Persistent vestibular examination abnormalities despite marked clinical improvement	Before vs. after erenumab	Clinical improvement occurred without changes in peripheral vestibular tests	Clinical improvement despite persistent vestibular abnormalities suggests a possible dissociation between symptomatic response and objective vestibular findings
Goto et al. (18)	Pure-Tone Audiometry, video Head Impulse Test (vHIT), posturography, clinical assessment	Bilateral low-frequency hearing loss with normal vHIT and posturography; diagnosis based on migraine-associated recurrent vertigo	Baseline clinical evaluation before treatment	Objective vestibular tests excluded major peripheral vestibular dysfunction while supporting the diagnosis of vestibular migraine	Comprehensive vestibular assessment aided diagnosis before initiating targeted therapy
Salmito et al. (17)	Comparison of 2001 and 2012 Vestibular Migraine Diagnostic Criteria; clinical assessment; headache and dizziness VAS	87.7% fulfilled the 2001 criteria compared with 77.8% fulfilling the 2012 criteria; the 2012 criteria were more specific while identifying fewer patients	2001 vs. 2012 diagnostic criteria	Comparison of diagnostic sensitivity/specificity and therapeutic response across diagnostic classifications	The 2012 Bárány/ICHD criteria improve diagnostic specificity without altering treatment response to prophylactic therapy.

### 3.4 Conventional pharmacological management

In most studies, conventional preventive treatments significantly improved vestibular migraine symptoms, although their effectiveness varied according to the medication class and clinical outcomes. Preventive therapies, including β-blockers, calcium-channel blockers, antidepressants, anticonvulsants, acetazolamide, nimodipine, cinnarizine/dimenhydrinate combinations, and botulinum toxin A, were associated with reductions in vertigo frequency, headache burden, dizziness-related disability, and migraine-related disability (11, 12, 15, 23, 24, 27, 29).

In comparative studies, no single conventional preventive therapy consistently demonstrated superiority over the others. Both venlafaxine and propranolol produced comparable improvements in vertigo severity and disability, while venlafaxine was additionally associated with improvements in depressive symptoms (15). Likewise, physician-selected preventive therapies including acetazolamide, amitriptyline, flunarizine, propranolol, and topiramate significantly reduced vestibular symptom severity, headache severity, and attack frequency, with no significant differences observed among treatment groups (29). Topiramate was effective at both 50 mg/day and 100 mg/day; however, the lower dose demonstrated a more favorable tolerability profile (11).

Several interventions demonstrated particular efficacy in improving vestibular symptoms. Flunarizine was more effective than symptomatic treatment alone in reducing the frequency and severity of vertigo (23), whereas combined therapy with cinnarizine and dimenhydrinate resulted in greater reductions in the frequency of both vertigo and headache attacks compared to lifestyle modification alone (24). Similarly, the addition of botulinum toxin A to oral preventive therapy resulted in further reductions in migraine-related disability and vertigo frequency compared to oral medication alone (12).

Despite the overall favorable effects of conventional preventive therapies, treatment response varied across patient populations. Conventional preventive medications showed limited efficacy in patients with refractory vestibular migraine before initiation of anti-CGRP therapy, while acute treatment with rizatriptan did not produce clinically meaningful improvements in vertigo, although modest improvements in headache-related symptoms were reported (26, 28) (Table 4A).

**Table 4:** Summary of studies reporting therapeutic outcomes.

A. Conventional management						
Study	Conventional therapy	Comparator	Treatment duration	Main outcomes	Main findings	Author's conclusion
Lovato et al. (28)	Propranolol, Flunarizine, Valproic acid	Baseline and subsequent anti-CGRP therapy	Mean 28.3 weeks	DHI, migraine days, videonystag -mography	No significant improvement in DHI, migraine frequency, or vestibular findings	Conventional preventive therapy showed limited effectiveness in this refractory population
Goto et al. (18)	Amitriptyline with Kampo medicine	Before galcanezumab	4 months	Dizziness, migraine frequency, hyperacusis	Dizziness and migraine frequency improved, whereas hyperacusis persisted	Conventional therapy provided only partial symptom control
Choi et al. (27)	Nimodipine, Topiramate, Combination therapy	Three treatment groups	3 months	Headache days, DHI, UCLA-DQ, dizziness days, MIDAS, HIT-6	All groups significantly improved headache and vestibular outcomes; no significant differences in dizziness outcomes among therapies	All preventive regimens were effective, with topiramate showing greater improvement in some headache-related outcomes
Staab et al. (26)	Rizatriptan	Placebo	Acute treatment of up to three attacks	Vertigo, dizziness, headache, quality of life	No significant benefit for vertigo at 1 h; modest benefit for headache-related symptoms at 24 h	Rizatriptan had limited efficacy for acute vestibular migraine attacks
Salviz et al. (15)	Venlafaxine; Propranolol	Venlafaxine vs. propranolol	4 months	DHI, VSS, vertigo frequency, anxiety, depression	Both therapies significantly reduced DHI, VSS, and vertigo attacks with no significant difference between groups; venlafaxine produced greater improvement in depressive symptoms	Venlafaxine and propranolol were similarly effective for vestibular migraine prophylaxis, while venlafaxine additionally improved depressive symptoms
Lepcha et al. (23)	Flunarizine plus betahistine	Betahistine with vestibular exercises	12 weeks	Vertigo frequency, vertigo severity, headache frequency, headache severity	Flunarizine significantly reduced vertigo frequency and severity compared with control, while headache outcomes were not significantly different	Flunarizine was effective in improving vestibular symptoms in migrainous vertigo
Gode et al. (11)	Topiramate (50 or 100 mg/day)	Low-dose vs. high-dose topiramate	6 months	Vertigo frequency, vertigo severity, headache frequency, headache severity	Significant reductions in vertigo and headache frequency and severity occurred in both groups; no efficacy difference between doses; adverse effects were more frequent with 100 mg/day	Topiramate was effective for vestibular migraine prophylaxis, with 50 mg/day providing a better tolerability profile
Gorur et al. (12)	Propranolol, flunarizine, or amitriptyline with or without botulinum toxin A	Oral therapy alone vs. oral therapy plus botulinum toxin A	NR	DHI, MIDAS, vertigo frequency	Both groups improved significantly; botulinum toxin A produced greater reductions in migraine disability and vertigo frequency, while oral medication type did not significantly influence outcomes	Botulinum toxin A enhanced treatment response when added to standard oral prophylaxis, particularly for headache severity and attack frequency
Teggi et al. (24)	Cinnarizine 20 mg + Dimenhydrinate 40 mg (Arlevertan®)	Lifestyle modification alone	6 months	Vertigo attacks, headache attacks	Vertigo attacks decreased from 5.3 to 2.1 and headache attacks from 4.3 to 1.7; 68% achieved ≥50% reduction in vertigo compared with 18% in controls	Arlevertan® significantly reduced vestibular migraine attacks and headache frequency compared with lifestyle measures alone.
Dominguez-Duran et al. (29)	Acetazolamide, Amitriptyline, Flunarizine, Propranolol, Topiramate	Comparison among prophylactic medications	5 weeks	Vestibular symptoms, headache intensity, monthly crises	Significant reductions in vestibular symptom intensity (−45.8 VAS points), headache intensity (−47.8 VAS points), and monthly crises (−15.6/month); no differences between medications	Preventive therapy significantly improved vestibular migraine symptoms regardless of the prophylactic medication selected.
Salmito et al. (17)	Antidepressants, anticonvulsants, calcium-channel blockers, β-blockers	Pre- vs. post-treatment	NR	Headache VAS, dizziness VAS	Significant improvement in headache and dizziness scores after prophylaxis irrespective of the diagnostic criteria used	Preventive therapy was effective regardless of whether patients fulfilled the 2001 or 2012 vestibular migraine criteria.
**B. CGRP-Targeted Therapies**						
**Study**	**Anti-CGRP therapy**	**Study design**	**Sample size**	**Main outcomes**	**Key results**	**Author's conclusion**
Russo et al. (16)	Erenumab, Fremanezumab, Galcanezumab	Prospective cohort	50	Vertigo, headache, MIDAS	90% achieved ≥50% reduction in vertigo frequency; dizziness days decreased from 10.3 to 0.8/month; 86% achieved ≥50% headache reduction	Anti-CGRP therapy produced substantial and sustained improvement in vestibular migraine
Hoskin et al. (10)	Erenumab, Fremanezumab, Galcanezumab, Ubrogepant	Retrospective cohort	28	Patient-reported improvement	21/25 improved; 15 had moderate-to-significant improvement	Anti- CGRP medications appeared beneficial for vestibular migraine
Lovato et al. (28)	Erenumab	Retrospective comparative study	23	DHI, migraine days, VNG	Significant improvements in DHI, migraine days, and VNG abnormalities; superior to conventional therapy	Anti-CGRP therapy was more effective than previous conventional preventive treatments
Inui et al. (13)	Erenumab	Case report	1	DHI, MIDAS, HIT-6, vestibular testing	Marked symptomatic improvement despite unchanged vestibular examinations and MRI	Symptomatic improvement occurred despite unchanged vestibular examinations and MRI
Kwon et al. (9)	Galcanezumab, Fremanezumab	Prospective observational study	20	KDHI, monthly dizziness days, VAS, PGIC	KDHI decreased from 43.3 to 22.6; dizziness days decreased from 20.8 to 6.7/month; 75% reported marked improvement	Both galcanezumab and fremanezumab were effective and well tolerated
Ihara et al. (25)	Erenumab	Retrospective observational study	19	Monthly migraine days, responder rate, migraine-associated symptoms, satisfaction	Monthly migraine days decreased by 6.6 at 3 months; 42% achieved ≥50% responder rate; improvement in photophobia, phonophobia, nausea, and high patient satisfaction	Erenumab was effective and well tolerated in routine clinical practice
Sharon et al. (14)	Galcanezumab	Randomized placebo-controlled trial	38	VM-PATHI, DHI, definitely dizzy days	Significant improvements in VM-PATHI, DHI, and dizzy days compared with placebo; no serious adverse events	Galcanezumab effectively improved vestibular migraine symptoms
Goto et al. (18)	Galcanezumab	Case report	1	Hyperacusis, HIT-6, migraine frequency	Marked improvement in hyperacusis, migraine severity, and overall symptoms after six months	Galcanezumab may improve auditory symptoms associated with vestibular migraine in addition to headache control
Shao et al. (19)	Rimegepant	Multicenter prospective self-controlled cohort	33	Vestibular symptom days, headache days, DHI, VSS-SF, HIT-6, GAD-7, PHQ-9, ABC	Vestibular symptom days decreased by 8.48/month and headache days by 5.88/month; 63.6% achieved ≥50% reduction in vestibular symptoms and 70.4% achieved ≥50% reduction in headache days; significant improvements in vestibular disability, psychological status, quality of life, and hearing were observed	Preventive rimegepant produced rapid and clinically meaningful improvements in vestibular migraine symptoms, psychological wellbeing, and quality of life
Chu et al. (20)	Rimegepant	Prospective single-arm clinical study	18	Acute vestibular symptoms, headache, VAS, adverse events	Significant reduction in vertigo, dizziness, nausea, photophobia/phonophobia, and headache beginning within 2 h; nearly complete symptom freedom achieved by 24–48 h; no adverse events reported	Rimegepant demonstrated rapid and effective relief of acute vestibular migraine attacks with excellent tolerability
Li et al. (22)	Rimegepant	Retrospective cohort study	86	2-h vertigo relief, vertigo NRS, predictors of response	2-h vertigo relief occurred in 76.2% of treated attacks; mean vertigo NRS improved by 4.44 points; younger age and better hearing predicted greater treatment response; no treatment-related adverse events observed	Rimegepant was highly effective for acute vestibular migraine, with patient age and hearing status influencing treatment response

### 3.5 CGRP-targeted therapies

Evidence for CGRP-targeted therapies was derived from randomized placebo-controlled evidence together with prospective observational studies, retrospective cohorts, real-world studies, and case reports. The randomized placebo-controlled trial demonstrated significant improvement with galcanezumab compared with placebo in vestibular migraine outcomes (14). Supportive observational evidence also reported clinically meaningful improvements with erenumab, fremanezumab, galcanezumab, rimegepant, and ubrogepant, including reductions in vertigo frequency, monthly dizziness days, headache burden, migraine-related disability, and patient-reported symptom severity (9, 10, 14, 16, 19, 20, 22, 28).

Clinical improvement was consistently observed across multiple outcome measures. Significant reductions in vertigo frequency, monthly dizziness days, and headache frequency were reported, with a substantial proportion of patients achieving a ≥50% reduction in symptoms. Preventive treatment with rimegepant demonstrated an early onset of efficacy, with significant improvements evident within two weeks of initiating therapy and sustained benefits after four weeks, including reductions in vestibular symptoms, headache frequency, vestibular disability, psychological distress, and improvements in quality of life. Additional improvements were observed in validated outcome measures, including the Dizziness Handicap Inventory (DHI), Korean Dizziness Handicap Inventory (KDHI), Migraine Disability Assessment (MIDAS), Vestibular Migraine Patient Assessment Tool and Handicap Inventory (VM-PATHI), migraine-associated symptoms, and Patient Global Impression scores. Randomized controlled evidence demonstrated that galcanezumab was superior to placebo in reducing dizziness-related disability and vestibular migraine symptoms (14). In addition, a retrospective comparative study reported greater clinical improvement following anti-CGRP therapy than during previous conventional preventive treatment. However, given the non-randomized nature of this comparison, this finding does not establish superiority of CGRP-targeted therapies over conventional preventive treatments (9, 14, 16, 19, 20, 22, 28).

In contrast, studies evaluating acute treatment demonstrated a much more rapid therapeutic effect. When rimegepant was administered during an acute vestibular migraine attack, improvements in vertigo, dizziness, nausea, photophobia, phonophobia, and headache were observed within two hours, with most patients achieving near-complete symptom resolution within 24–48 h. Retrospective analyses additionally demonstrated that approximately three-quarters of treated vestibular migraine attacks achieved meaningful vertigo relief within 2 h, with younger age, lower baseline symptom severity, and better hearing associated with greater treatment response (20, 22). These findings were further supported by real-world observational data, which showed clinically meaningful reductions in monthly migraine days, high responder rates, high patient satisfaction, and acceptable safety profiles in real-world clinical practice (10, 25). Case reports also indicated that galcanezumab could have a beneficial effect on symptoms of auditory involvement (hyperacusis and tinnitus) associated with vestibular migraine, and erenumab had a significant effect on headache and dizziness symptoms despite persistent abnormalities in vestibular testing and imaging, indicating that symptomatic improvement may occur independently of normalization of objective vestibular findings and suggesting a potential dissociation between clinical response and measurable peripheral vestibular dysfunction. These findings support further investigation into the central vestibular and multisensory pathways through which CGRP-targeted therapies may exert their clinical effects (13, 18) (Table 4B).

## 4 Discussion

The current systematic review provides an overview of the available evidence on the diagnosis and pharmacological treatment of vestibular migraine, including conventional preventive therapies and emerging treatments targeting calcitonin gene-related peptide (CGRP). Overall, the included studies indicate that VM remains primarily a clinical diagnosis supported by targeted vestibular, audiological, and radiological investigations, and that several preventive therapies have demonstrated or been associated with improvements in vestibular and migraine-related outcomes. CGRP-targeted therapies showed promising improvements across several clinical outcomes, with an expanding evidence base encompassing randomized and observational studies.

The population characteristics of the included studies were broadly consistent with the known epidemiology of VM. The predominance of females with migraine disorders is well known, and the majority of adult participants were women in their fourth or fifth decade of life. Epidemiological studies conducted previously have reported that VM occurs in women about 2–4 times more often than men, which may be due to a combination of hormonal factors, genetic susceptibility, and shared migraine pathophysiology (30, 31). Population-based studies have also shown that the highest incidence of VM is in middle adulthood, which is reflected in the age distribution of the adult populations included in this review (5, 32). The evidence bases also included a large pediatric multicenter study, highlighting that migraine-associated vestibular manifestations extend across age groups and that age-specific clinical characteristics should be considered when interpreting the available literature.

One of the major findings of this review is that the diagnosis of vestibular migraine continues to rely primarily on clinical diagnostic criteria rather than validated diagnostic tests. Vestibular migraine is fundamentally a clinical diagnosis based on symptom history and the Bárány Society and International Classification of Headache Disorders (ICHD-3) criteria, while neuroimaging and vestibular laboratory studies are mainly used to rule out other vestibular disorders. At present, no vestibular test, audiological measure, imaging finding, or biological marker has sufficient validated diagnostic accuracy to serve as an independent diagnostic biomarker for VM. This is in line with current international consensus statements, which emphasize that no single vestibular test or imaging modality has sufficient sensitivity or specificity to independently confirm VM (33).

Objective vestibular abnormalities may persist despite substantial clinical improvement, as shown by the included diagnostic studies. These results have been previously reported in other studies and indicate that vestibular function tests may not be correlated with symptom severity or treatment response and may be abnormal between attacks (34). The persistence of vestibular abnormalities despite symptomatic improvement may suggest a greater contribution of central vestibular and multisensory mechanisms to the clinical response to CGRP inhibition, rather than improvement resulting solely from normalization of peripheral vestibular function (35). This clinical–physiological dissociation supports a potential central component of CGRP-targeted therapeutic effects in VM, although the relative contributions of central and peripheral mechanisms remain to be clarified.

An important diagnostic challenge is the substantial clinical overlap between VM and other vestibular disorders, particularly Ménière's disease (MD), benign paroxysmal positional vertigo (BPPV), and persistent postural-perceptual dizziness (PPPD). VM and MD may both present with recurrent vertigo, tinnitus, and aural fullness; however, documented fluctuating sensorineural hearing loss, particularly at low-to-medium frequencies, favors MD. Distinguishing these disorders may nevertheless be difficult, particularly early in their clinical course or when auditory symptoms accompany VM. BPPV is generally characterized by brief episodes of positionally triggered vertigo associated with characteristic positional nystagmus, whereas positional vertigo may also occur in VM. PPPD is distinguished by a persistent pattern of dizziness or unsteadiness, typically exacerbated by upright posture, movement, or complex visual stimuli, although PPPD may coexist with VM. These areas of overlap emphasize the importance of careful symptom characterization, audiological assessment, positional examination, migraine history, and longitudinal clinical evaluation when establishing the diagnosis (6, 33, 36).

The biological overlap between VM and MD may further contribute to their diagnostic complexity. Flook et al. demonstrated differences in proinflammatory cytokine profiles between patients with VM and MD, suggesting that despite overlapping clinical manifestations, the two conditions may involve distinct inflammatory mechanisms (37). More recently, Cruz-Granados et al. identified distinct immune profiles using single-cell multiomic approaches, providing further evidence of potentially different biological mechanisms underlying these clinically overlapping disorders (38). These findings are relevant to the search for objective biomarkers capable of distinguishing VM from MD; however, such biomarkers remain investigational and are not currently established for routine clinical diagnosis. Similarly, although neuroimaging studies have identified alterations involving vestibular and multisensory processing networks in VM, no imaging finding has yet demonstrated sufficient diagnostic accuracy for independent clinical confirmation. Vestibular testing and imaging should therefore remain complementary to established clinical criteria, primarily assisting in the exclusion of alternative diagnoses and characterization of associated vestibular dysfunction.

In terms of conventional drug therapy, the present review showed that several drugs were associated with reductions in the frequency of vertigo, headache burden, and disability due to dizziness: β-blockers, calcium-channel blockers, anticonvulsants, antidepressants, acetazolamide, and flunarizine. The results are in line with the previous systematic reviews that reported moderate evidence for using conventional migraine prophylactic drugs in VM, despite the lack of large randomized trials (39, 40). Treatment selection is therefore generally individualized according to patient characteristics, comorbidities, contraindications, and tolerability rather than an established superiority of one conventional preventive therapy over another.

The comparable clinical benefits reported with several conventional preventive medications is also supported by previous comparative studies. Propranolol, venlafaxine, flunarizine, and topiramate have each been associated with reductions in vertigo attacks and headache burden. The variability in therapeutic response and tolerability across these agents further supports an individualized approach to preventive treatment, based on patient characteristics, adverse-effect profile, psychiatric comorbidities, and cardiovascular contraindications.

CGRP-targeted therapies represent an important emerging therapeutic option for VM. Across the included studies, Erenumab, fremanezumab, galcanezumab, rimegepant, and ubrogepant were associated with improvements in vertigo frequency, dizziness-related disability, headache frequency and migraine symptoms, with generally favorable tolerability. The therapeutic evidence includes placebo-controlled randomized data for galcanezumab (14), complemented by prospective and real-world studies reporting clinical improvements with other CGRP-targeted agents (9, 10, 13, 16, 18–20, 22, 25, 28). Together, these findings provide a consistent and encouraging therapeutic signal across different CGRP-targeted approaches. Recent studies evaluating rimegepant also reported potential benefit in both preventive and acute treatment settings, with rapid symptom improvement, significant reductions in vestibular symptom frequency and headache burden, improvements in psychological wellbeing and balance confidence, and favorable response rates in routine clinical practice as early as two weeks for preventive therapy and within 2 h for acute treatment (19, 20, 22). These results are consistent with the emerging evidence linking CGRP to VM pathophysiology (41, 42). Experimental research has shown CGRP and its receptors within vestibular, trigeminovascular, and cerebellar pathways, providing a biologically plausible mechanism by which CGRP inhibition could be beneficial for both migraine and vestibular manifestations (43).

The inclusion of randomized evidence in this review further supports the therapeutic potential of anti-CGRP therapy The clinical improvements demonstrated with galcanezumab compared with placebo (14) complement the favorable therapeutic responses reported across observational and real-world studies and are consistent with the broader emerging evidence supporting CGRP-directed treatment in VM (44). Nevertheless, direct randomized comparisons with conventional preventive therapies remain limited, and current evidence does not establish the comparative superiority of CGRP-targeted treatment.

It is also worth noting that symptomatic improvement following anti-CGRP therapy may occur despite persistent abnormalities on vestibular physiological testing. This clinical–physiological dissociation is compatible with neurophysiological models proposing an important role for central vestibular and multisensory processing in VM and suggests that the therapeutic effects of CGRP inhibition may extend beyond normalization of peripheral vestibular function (45, 46). Functional neuroimaging studies demonstrating altered activation of vestibular cortical networks and cerebellar pathways further support the biological plausibility of central involvement in VM (47), although the relative contributions of central and peripheral mechanisms to treatment response remain to be established.

The safety results were generally favorable for both conventional and anti-CGRP treatments. Conventional preventive medications had adverse-effect profiles consistent with their respective pharmacological classes, while CGRP-targeted therapies were well tolerated with few serious adverse events. These findings are similar to those found in large trials of CGRP monoclonal antibodies, which have shown favorable long-term safety, low discontinuation rates, and high patient adherence relative to several traditional preventive therapies (48, 49).

A major strength of this systematic review is its comprehensive and contemporary evaluation of VM, integrating diagnostic approaches with both established pharmacological treatments and emerging CGRP-targeted therapies. This combined approach provides a clinically relevant perspective on how diagnostic challenges may influence treatment selection and interpretation of therapeutic outcomes. The inclusion of recent evidence on CGRP-targeted therapies further allows the review to capture important developments in the rapidly evolving management of VM.

Certain limitations should be acknowledged. The available evidence was characterized by substantial clinical and methodological heterogeneity in study populations, interventions, outcome measures, and follow-up periods. Many studies were observational and involved relatively small samples, while randomized controlled trials and direct head-to-head comparisons between conventional and CGRP-targeted therapies remained limited. These factors restricted direct comparisons between treatment strategies and prevented meaningful quantitative synthesis. In addition, potential publication bias cannot be excluded, particularly in the emerging literature on newer therapies. The inability to retrieve some potentially eligible reports may also have introduced selection bias. Further studies using standardized VM diagnostic criteria, therapeutic regimens, follow-up intervals, and validated VM-specific outcome measures are needed to improve comparability across studies and strengthen future quantitative and comparative analyses.

## 5 Conclusion

Vestibular migraine remains a clinically challenging disorder in which diagnosis relies primarily on established clinical criteria and treatment requires an individualized approach. Current evidence supports established preventive therapies, while CGRP-targeted treatments represent a promising therapeutic option with benefits across vestibular and migraine-related outcomes. The growing evidence for these therapies may broaden treatment options, particularly for patients with persistent symptoms. Further high-quality studies are needed to clarify their optimal role and support more standardized, evidence-based management of VM.
